# Comparison of the Regulatory Effects of Host Factors on Viral Internal Ribosomal Entry Sites

**DOI:** 10.3390/vetsci12121128

**Published:** 2025-11-27

**Authors:** Rupaly Akhter, Kazi Anowar Hossain, Bouchra Kitab, Mohammad Enamul Hoque Kayesh, Kyoko Tsukiyama-Kohara

**Affiliations:** 1 Transboundary Animal Diseases Center, Joint Faculty of Veterinary Medicine, Kagoshima University, Kagoshima 890-0065, Japan; rupaly_akhter@yahoo.com (R.A.); kazianowar29@gmail.com (K.A.H.); bouchra.kitab17@gmail.com (B.K.); mehkayesh@pstu.ac.bd (M.E.H.K.); 2 Department of Pharmacology & Toxicology, Faculty of Animal Science and Veterinary Medicine, Sher-e-Bangla Agricultural University, Sher-e-Bangla Nagar, Dhaka 1207, Bangladesh; 3 Department of Microbiology and Public Health, Faculty of Animal Science and Veterinary Medicine, Patuakhali Science and Technology University, Barishal 8210, Bangladesh

**Keywords:** internal ribosome entry site (IRES), polycystic kidney disease 1-like 3 (PKD1L3), ubiquitin-specific peptidase 31 (USP31), foot-and-mouth disease virus (FMDV), classical swine fever virus (CSFV), hepatitis C virus (HCV), encephalomyocarditis virus (EMCV), dengue virus (DENV)

## Abstract

Host factors play critical roles in viral IRES-mediated translation by modulating the efficiency and specificity of viral protein synthesis. In this study, we used small interfering RNA (siRNA) treatment to silence and plasmid-based expression to overexpress PKD1L3 and USP31. Silencing *PKD1L3* and *USP31* suppressed IRES activity in FMDV and CSFV RNAs, whereas the overexpression of PKD1L3 did not have a significant effect, and USP31 overexpression resulted in only a modest increase in CSFV-IRES activity. Silencing *PKD1L3* significantly reduced EMCV-IRES activity but had no significant effect on HCV- or DENV-IRES activity, and silencing *USP31* had no significant effect on the activities of these three IRESs. Notably, the combined overexpression of PKD1L3 and USP31 significantly suppressed HCV-IRES activity, suggesting potential context-dependent interactions. These findings indicated that PKD1L3 and USP31 contribute more prominently to CSFV-, FMDV-, and EMCV-IRES-mediated translation than to HCV- or DENV-IRES-driven translation. Collectively, our results provide new insights into the host factors involved in IRES-mediated viral translation, establish a foundation for future in vivo studies to elucidate the specific roles of PKD1L3 and USP31 during viral infection, and indicate potential strategies for mitigating these viruses.

## Simple Summary

Host factors are essential for viral internal ribosomal entry site (IRES)-mediated translation and influence the efficiency and specificity of viral protein synthesis. We previously identified two host factors, polycystic kidney disease 1-like 3 (PKD1L3) and ubiquitin-specific peptidase 31 (USP31), as contributors to the IRES-mediated translation of foot-and-mouth disease virus (FMDV) and classical swine fever virus (CSFV). In this study, we confirmed their roles in FMDV and CSFV processes and explored their potential effect on other viral IRESs, including those of encephalomyocarditis virus (EMCV), hepatitis C virus (HCV), and dengue virus (DENV). Our results showed that PKD1L3 and USP31 support CSFV-, FMDV-, and EMCV-IRES-mediated translation, with limited effects on HCV and DENV IRESs. These findings provide new insights into the host factors that regulate viral translation and establish a foundation for future in vivo studies to define their roles during viral infection and develop strategies for virus mitigation.

## 1. Introduction

Some viral RNAs exploit an internal ribosomal entry site (IRES) to initiate cap-independent translation by directly recruiting ribosomes to the internal region of the messenger RNA (mRNA) [1]. These highly structured elements are typically located within the 5′ untranslated regions (5′UTRs) of viral mRNAs and some eukaryotic mRNAs [1]. Based on their secondary structure, length, required proteins, and mechanism of action, picornavirus IRESs are classified into five types: poliovirus (Type 1), foot-and-mouth disease virus (FMDV) (Type II), hepatitis A virus (Type III), hepatitis C virus (HCV)-like viruses (Type IV), and Aichivirus-like viruses (Type V) [2]. The IRES mechanisms may vary between viruses; for example, an IRES element is present within the 5′UTR of the FMDV RNA genome [3,4], whereas other picornaviruses, including poliovirus and encephalomyocarditis virus (EMCV), and flaviviruses, including HCV, have virus-specific IRES elements in their 5′UTRs [5,6,7].

Each type of IRES has evolved distinct mechanisms and specific factor requirements for ribosome recruitment. Their horizontal transfer among viruses underscores their crucial role in viral evolution, host specificity, and pathogenesis [8]. By initiating translation independently of the 5′ cap, viral IRES elements enable viruses to bypass host regulatory controls [9,10]. IRES-mediated translation is a key mechanism for viral propagation and a promising target for antiviral research [1]. Mechanisms underlying IRES-mediated translation vary among viruses. For example, the RNA genome of FMDV contains an IRES element within the 5′UTR that requires specific host factors for ribosome recruitment [3,4]. Other picornaviruses, such as poliovirus and EMCV, as well as flaviviruses including HCV, possess structurally distinct IRES elements in their 5′UTRs with differing dependencies on host factors for translation initiation [5,6]. Despite these variations, most viruses rely on host-derived proteins for efficient IRES-driven translation [11]. Therefore, identifying these host factors is essential for understanding viral replication mechanisms and may reveal novel targets for antiviral interventions. Host factors, including eukaryotic initiation factors (eIFs) and RNA-binding proteins (RBPs), such as IRES-transacting factors (ITAFs), play a critical role in modulating IRES activity by facilitating the recruitment of the translation machinery and regulating internal initiation, particularly under stress conditions such as viral infection [12,13,14,15,16,17]. In addition to these well-known proteins, other host factors may influence IRES-driven translation. Recent studies have identified two eukaryotic proteins that have received attention as potential host factors for viral IRESs: polycystic kidney disease 1-like 3 (PKD1L3) and ubiquitin-specific peptidase 31 (USP31). Interest in these proteins was sparked when the French maritime pine extract, pycnogenol^®^ (PYC), was shown to both suppress IRES activity and downregulate *PKD1L3* and *USP31* expression in research on FMDV and classical swine fever virus (CSFV) [18]. Following up on that report, we further investigated the possible participation of PKD1L3 and USP31 in IRES-mediated translation in FMDV and CSFV using bicistronic plasmid reporter assays and found further evidence that these proteins may be host factors through their interactions with eIF3c and Hsp90β [18,19]; however, caution should be used when interpreting the former information [20].

Research on PKD1L3 and USP31 as host factors exploited by IRESs is limited to FMDV and CSFV; however, there are reasons to believe that they could play similar roles in the translation of other viruses, which might contribute to cross-IRES utilization. We previously performed a comprehensive analysis of host factors for which gene expression is modified in response to PYC treatment using microarray analyses [18], which showed that PYC has antiviral effects against HCV [21] and dengue virus (DENV) [22], similar to its reported effects on FMDV and CSFV [18]. Given the similar sensitivity of these viruses to PYC treatment, it is possible that similarities may also exist in the host factors exploited by their IRES-mediated translation mechanisms; however, there are no reported data on the potential host factor roles of PKD1L3 and USP31, other than those reported for FMDV and CSFV.

Against this background, we aimed to further corroborate the roles played by the host factors PKD1L3 and/or USP31 in FMDV and CSFV infection and investigate whether these proteins similarly participate in the IRES-mediated translation of HCV, DENV, and EMCV using bicistronic reporter assays to ascertain the effects of protein silencing and overexpression. Bicistronic reporter assays have a number of known advantages and disadvantages regarding their use [20]. A commonly reported disadvantage is the potential for false-positive IRES activity caused by cryptic promoter and/or splicing activities, particularly in the case of cryptic promoter activity in HCV IRES [23]. Critical control experiments of the authenticity and stability of bicistronic mRNAs and careful interpretation are required to rule out such confounding effects and validate the IRES activity. Therefore, we have also addressed the assay integrity controls, examining RNA transcripts and promoterless RNA, and examined the role of PKD1L3 and USP31 through cross-IRES examination.

## 2. Materials and Methods

### 2.1. Cells

Human embryonic kidney (HEK)293 cells were obtained from the American Type Culture Collection (ATCC): an HEK-derived FMDV-IRES-expressing cell line (B10) that was established previously [13] and an HEK-derived CSFV-IRES-expressing cell line (pCI5) that was established previously [24], and HEK293 cells that can support these IRES activities [25]. Porcine-derived SKL cells were kindly provided by Dr. Sakoda, Hokkaido University, and used as previously described [18]. pRF vectors containing the FMDV IRES (serotype C) [26], EMCV IRES [27] (pRE), and HCV IRES [6] (pRH) were gifted by Dr. Kensuke Hirasawa (Memorial University of Newfoundland) and Dr. Sung-Key Jang (Pohang University of Science and Technology), and pRF vectors containing the CSFV IRES were gifted by Professor Graham J. Belsham (University of Copenhagen). DENV IRES was constructed using nucleotide No.1-160 of DENV2C (gifted by Dr. Michinori Kohara, Tokyo Metropolitan Institute of Medical Science), as described previously [28]. HEK293-based cells were cultured in Dulbecco’s modified Eagle’s medium (Nissui, Tokyo, Japan), including 10% fetal calf serum (Thermo Fisher Scientific Co., Waltman, MA, USA) and glutamine. All cells were maintained at 37 °C with 5% CO_2_.

### 2.2. Plasmid Transfection and Measurement of IRES Activity

Plasmid transfection, IRES activity measurements, and cell viability assessments were performed as described previously [18]. B10 and PCI5 cell lines were used to evaluate FMDV- and CSFV-IRES activities, respectively. To assess other viral IRES activities, HEK293 cells were transfected with HCV-IRES-expressing bicistronic vector (pRH-IRES) [6], EMCV-IRES-expressing bicistronic vector (pRE-IRES) [27], or DENV-IRES-expressing vector (pRD-IRES) [22]. Briefly, cells were transfected with the relevant bicistronic IRES reporter plasmid (0.5 mg/well of a 96-well plate) using Lipofectamine LTX Reagent (ThermoFisher Scientific Inc., Waltman, MA, USA), incubated at 37 °C for 72 h, and the resultant culture was assessed for viability by performing a water-soluble tetrazolium salt-1 (WST-1) (Takara Bio., Shiga, Japan) assay and measuring the optical density at 450 nm (OD_450_). The results were calculated as the OD_450_ versus that of mock-transfected or control siRNA-treated cells. Luciferase assays were performed as previously described [19]. IRES activity was calculated by dividing the firefly luciferase activity by the *Renilla* luciferase activity. The results for IRES activity are indicated as the percentage of each value versus that of the relevant control siRNA or mock-transfected cells (none).

### 2.3. Detection, Quantitation, and Synthesis of RNA

The dicistronic RNA expression in FMDV-IRES-expressing cells (B10, B5, G7) and CSFV-IRES-expressing cells (pCI5) was detected using a digoxigenin (DIG)-labeled detection kit (DIF Northern Starter kit, Merck, Darmstadt, Germany) in a purified total RNA run on 1.2% formaldehyde agarose gels (1 mg/lane), as previously described [24].

The amount of dicistronic RNA in B10 and pCI5 cells was measured by RT-qPCR that targeted *Renilla* luciferase genes using the primers 5′-AGAACCAGAAGAATTTGCAG-3′ and 5′-TGGTAAATCATCACTTGCAC-3′, as previously described [18].

Dicistronic RNAs were synthesized from *Hpa*I-digested pRF-FMDV-IRES and pRF-CSFV-IRES plasmid DNA using the RiboMax Large Scale RNA Production System T7 (Promega), as described previously [19]. The synthesized RNA was transfected into cells using the TransIT mRNA transfection kit (Mirus Bio, Madison, WI, USA) following the manufacturer’s protocol.

### 2.4. siRNA Transfection and Rescue by Plasmid DNA Transfection

The siRNAs (5 nM) targeting *PKD1L3* and *USP31* were designed as described previously [10] (*PKD1L3* siRNA, 5′-CAGUUCAUGGUUUGCAAGCUCUUAA-3′; *USP31* siRNA, 5′- CAGCACAGCCGCGACUUCAAGACUA-3′). The control siRNA used was ON-Target Plus siRNA Control (Horizon/Dharmacon, Lafayette, CO, USA). A mock transfection was performed in the absence of siRNAs. These siRNAs (5 nM) were transfected via reverse transfection using Lipofectamine^TM^ RNAiMax Reagent (Invitrogen) according to the manufacturer’s protocol [10]. The efficiency of *PKD1L3* and *USP31* knockdown was confirmed by qRT-PCR (gene silencing efficiency measured by performing qRT-PCR was 80–90%) and Western blotting, as described previously [18,19,24].

The siRNA rescue experiment was performed by transfecting the plasmid DNA (5 mg/well in 24-well plates) using Lipofectamine LTX (Thermo Fisher Co.) reagent 24 h after siRNA transfection, following the manufacturer’s protocol.

### 2.5. Plasmid Construction and Sequencing

pRF-EGFP-PURO-EF1A-hPKD1L3-Myc and pRF-EGFP-PURO-EF1A-hUSP31-HA plasmids were constructed using VectorBuilder (Kanagawa, Japan). DNA sequencing was performed by Eurofins Japan (Tokyo, Japan), and the DNA sequences were characterized using GENETYX-Mac software version 22 (GENETYX Co., Tokyo, Japan) and GENBANK. Vector expression was confirmed by Western blotting or immunofluorescence analysis.

### 2.6. Immunofluorescence Analysis

The cells were fixed with 1% paraformaldehyde at room temperature for 10 min. After treatment with 1% triton-X100-PBS(-) at room temperature for 10 min, the samples were incubated with an anti-PKD1L3 rabbit antibody (ab234670, Abcam Co., Cambridge, UK) and anti-USP31 mouse monoclonal antibody (sc-100634, Santa Cruz Biotechnology, Inc., Dallas, TX, USA) at a 1 mg/mL IgG concentration with 1%BSA-PBS(-). Primary antibodies were detected using anti-rabbit Alexa Fluor 488 (Thermo Fisher Scientific) or anti-mouse Alexa Fluor 568 (Thermo Fisher Scientific). The stained cells were observed using a fluorescence microscope (BZ-X-700; Keyence Co., Osaka, Japan). The immunofluorescence images were quantified using ImageJ software (version 1.54p).

### 2.7. Statistics

All numerical data are presented as the mean  ±  standard deviation for experiments conducted in triplicate (n = 3), and data were prepared graphically using GraphPad Prism (version 9) software. Statistical significance was evaluated using Student’s *t*-test, one-way ANOVA, or Dunnett’s test, with *p* < 0.05 considered statistically significant.

## 3. Results

### 3.1. Confirmation of PKD1L3 and USP31 as Host Factors for FMDV and CSFV IRESs

**Figure 1:**
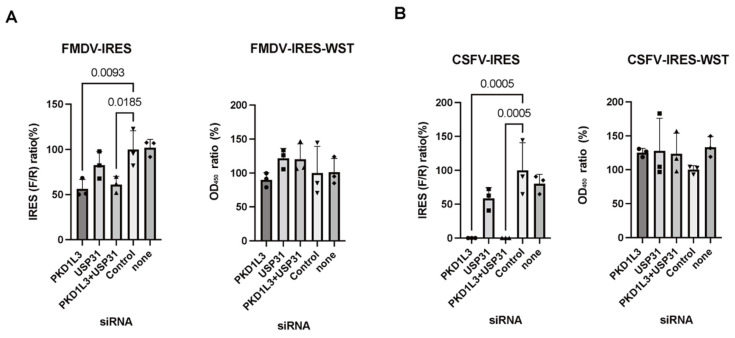
Examination of PKD1L3 and USP31 in foot-and-mouth disease virus (FMDV)- and classical swine fever virus (CSFV)-internal ribosomal entry site (IRES) activity using siRNA. (**A**) siRNAs targeting *PKD1L3* and *USP31* were transfected into FMDV-IRES-expressing cells (B10) (left). *p*-values less than 0.05, denoting significance, are shown. IRES activity was calculated as a percentage relative to that in cells treated with control siRNA. The cell viability after siRNA treatment was detected by performing a WST assay (right). The ratio of the OD_450_ value to that of the control siRNA-treated cells (%) is indicated. Error bars indicate standard deviations (SDs) (n = 3). (**B**) siRNAs targeting *PKD1L3* and *USP31* were transfected into CSFV-IRES-expressing cells (pCI5) (left). *p*-values less than 0.05 indicated significant effects. The viability of siRNA-treated cells was measured by performing a WST assay (right). The ratio relative to that in the cells treated with the vector control (%) is indicated. Error bars indicate SDs (n = 3). F/R, ratio of firefly luciferase activity to *Renilla* luciferase activity. All experiments were performed three times, and representative results are shown.

Previously, we found evidence implicating PKD1L3 and USP31 in FMDV-IRES activity [18] and CSFV-IRES activity [18]. To validate the ability of the system used in this study to detect the participation of PKD1L3 and USP31 in IRES-mediated translation, we investigated FMDV- and CSFV-IRES activity after *PKD1L3* and *USP31* silencing and overexpression. When IRES-expressing cells were transfected with siRNAs targeting PKD1L3 and/or USP31, both *PKD1L3* and *USP31* silencing significantly reduced FMDV (Figure 1A) and CSFV (Figure 1B) IRES activity. Silencing *USP31* alone produced absolute values indicative of changes in the same direction (reduced activity), but the differences were not statistically significant (Figure 1A,B).

**Figure 2:**
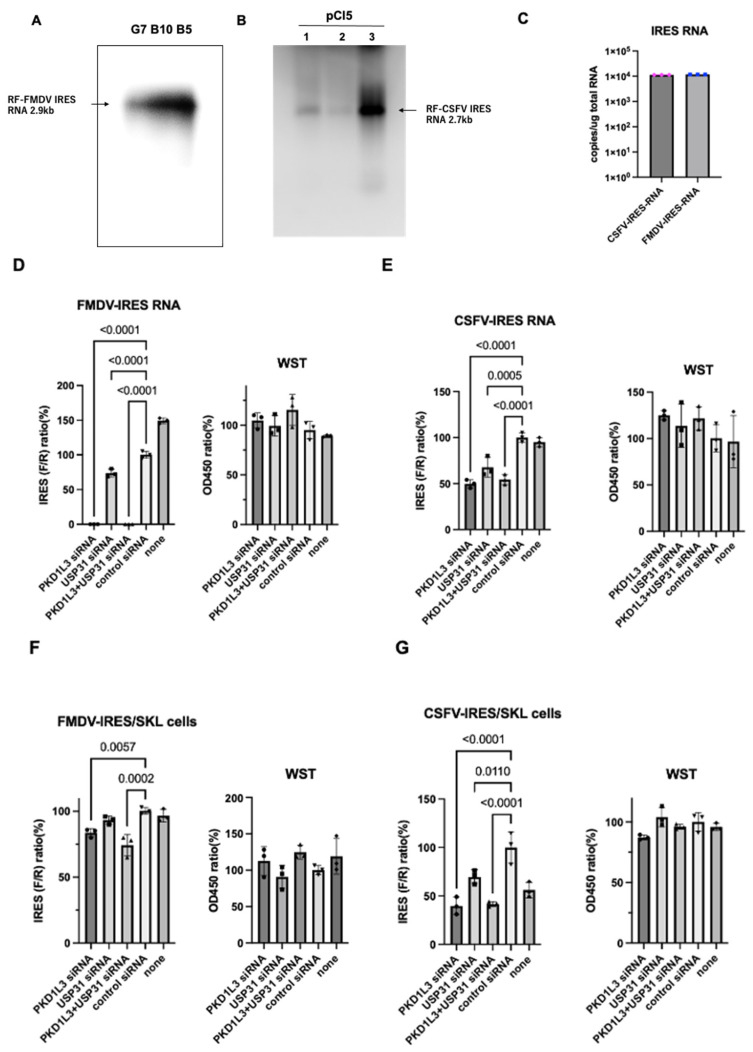
The assay integrity controls for the bicistronic IRES assay. (**A**) Validation of dicistronic RNA in FMDV-IRES-expressing cells and (**B**) CSFV-IRES-expressing cells using DIG-labeled *Renilla* luciferase RNA probes. (**C**) Quantification of dicistronic RNA in B10 and pCI5 (**D**) by qRT-PCR. (**D**) The dicistronic FMDV-IRES RNA and CSFV-IRES RNA (**E**) were transfected with or without siRNAs, and IRES activities were measured (left). Cell viability was measured by WST assay (right). FMDV-IRES (**F**) and CSFV-IRES (**G**) activities were analyzed with siRNAs using SKL cells (left). Cell viability was measured by WST assay (right). *p*-values less than 0.05, denoting significance, are shown. All experiments (n = 3) were performed three times, and representative results are shown.

To validate assay integrity, we examined the dicistronic RNAs in FMDV- and CSFV-IRES-expressing cells (Figure 2A,B), quantified these RNAs (Figure 2C), examined the effects of IRES using transcribed dicistronic RNAs (Figure 2D), and measured IRES activity using porcine-derived SKL cells (Figure 2F,G).

**Figure 3:**
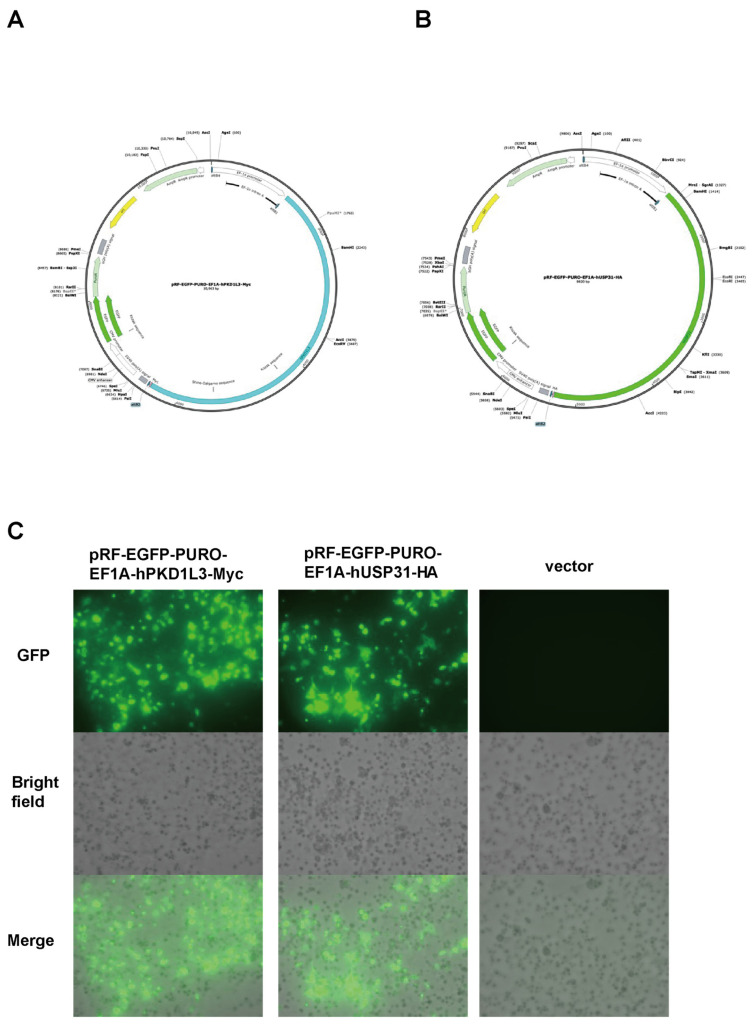
Structure of PKD1L3 and USP31 expression vector and examination under a fluorescence microscope. (**A**) Schematic representation of the pRF-PURO-EGFP-hPKD1L3-Myc plasmid DNA. (**B**) Schematic representation of the pRF-PURO-EGFP-hUSP31-HA plasmid DNA. (**C**) Fluorescence microscopy analysis of transfected cells showing GFP expression, confirming successful transfection. Images were acquired using a BZ-X700 microscope at 200× magnification.

We also investigated overexpression using specially constructed plasmids (pRF-PURO-EGFP-hPKD1L3-Myc expression vector and pRF-PURO-EGFP- hUSP31-HA expression vector; Figure 3A–C and Figure S1) and performed an immunofluorescence assay, which indicated the nuclear localization of PKD1L3 and cytoplasmic localization of USP31, as calculated based on Pearson’s correlation coefficient (Supplementary Figure S1A,B, Table S1).

**Figure 4:**
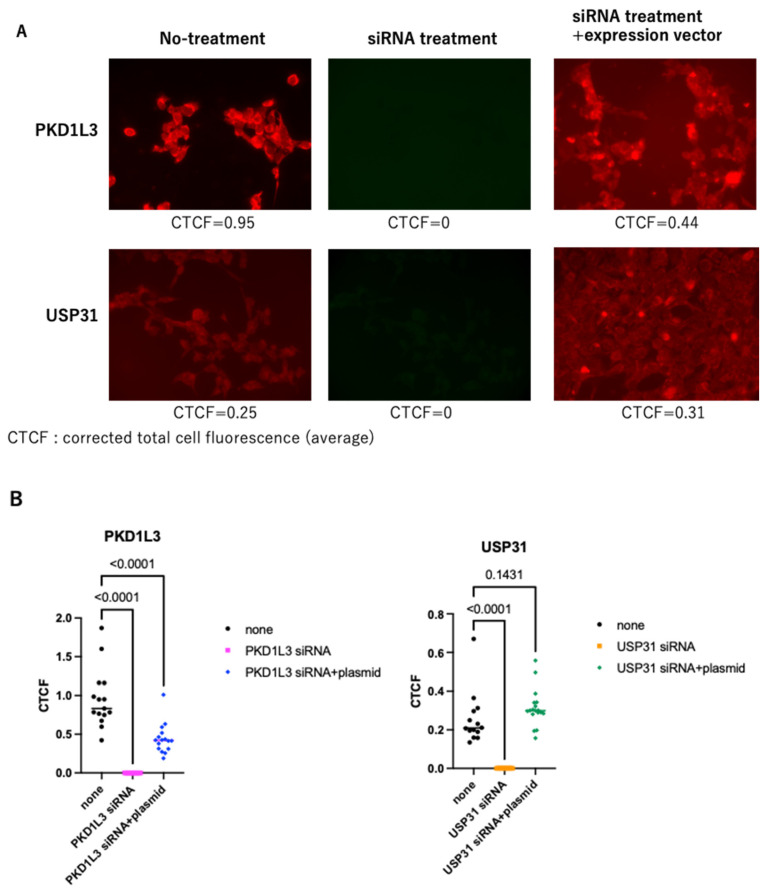
Validity of siRNAs and rescue by overexpression targeting PKD1L3 and USP31 was examined by immunofluorescent assay (×400). (**A**) Cells were treated with *PKD1L3* and *USP31* siRNA (5 nM) (middle upper and middle lower) and further transfected with expression plasmids for PKD1L3 and USP31 (right upper and lower). PKD1L3 and USP31 proteins were detected by the immunofluorescent assay, as described in the Methods section. Fluorescent images were quantified by ImageJ software and expressed as CTCF (corrected total cell fluorescence). Average CTCF values are also indicated below. (**B**) Dot plots presenting the quantitation of PKD1L3 and USP31 fluorescence signals measured as CTCF using ImageJ software. Statistical analysis was performed using one-way ANOVA and Dunnett’s analysis.

To validate the effect of siRNA and its rescue by plasmid overexpression, we transfected expression vectors after siRNA treatment (Figure 4A). ImageJ quantitative analysis showed that expression of PKD1L3 and USP31 was reduced by siRNA silencing (CTCF = 0) and recovered upon plasmid transfection to 46.3% with pRF-PURO-EGFP-hPKD1L3 and 124% with pRF-PURO-EGFP-hUSP31 (Figure 4B).

**Figure 5:**
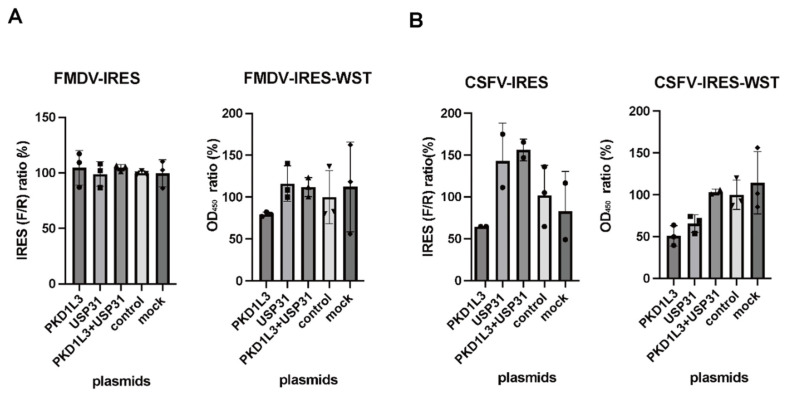
Effects of PKD1L3 and USP31 overexpression using an expression vector on foot-and-mouth disease virus (FMDV)- and classical swine fever virus (CSFV)-internal ribosomal entry site (IRES) activities. (**A**) PKD1L3 and/or USP31 were overexpressed through the transfection of pRF-PURO-EGFP-Myc and/or pRF-PURO-EGFP-hUSP31-HA plasmid DNA. FMDV-IRES activity (left) and cell viability (right) were measured relative to those in control vector-transduced cells. The ratio relative to that in the cells treated with the vector control (%) is indicated. Error bars are standard deviations (SDs; n = 3). (**B**) CSFV-IRES activity (left) and cell viability (right) were measured relative to those in control vector-transduced cells. The ratio relative to that in the cells treated with the vector control (%) is indicated. Error bars are SDs (n = 3). *p*-values less than 0.05, denoting significance, were not observed. F/R, ratio of firefly luciferase activity to *Renilla* luciferase activity. All experiments were performed three times, and representative results are shown.

Neither PKD1L3 nor USP31 overexpression significantly affected the FMDV-IRES activity (Figure 5A). Overexpression of USP31 alone and the combined overexpression of PKD1L3 and USP31 increased CSFV-IRES activity, whereas overexpression of PKD1L3 alone decreased CSFV-IRES activity, possibly because of its cytotoxicity and/or suppression mediated by an overdose (Figure 5B). Different doses of PKD1L3 or USP31 expression vectors showed similar effects to those of the control vector, indicating that overexpression did not have a significant effect on IRES RNA activity [19].

### 3.2. Investigation of PKD1L3 and USP31 as Host Factors for HCV, EMCV, and DENV IRESs

To ascertain the participation of PKD1L3 and USP31 in the IRES activity of the three other viruses (HCV, EMCV, and DENV), we investigated the effects of *PKD1L3* and *USP31* silencing and overexpression on the activity of each relevant IRES.

**Figure 6:**
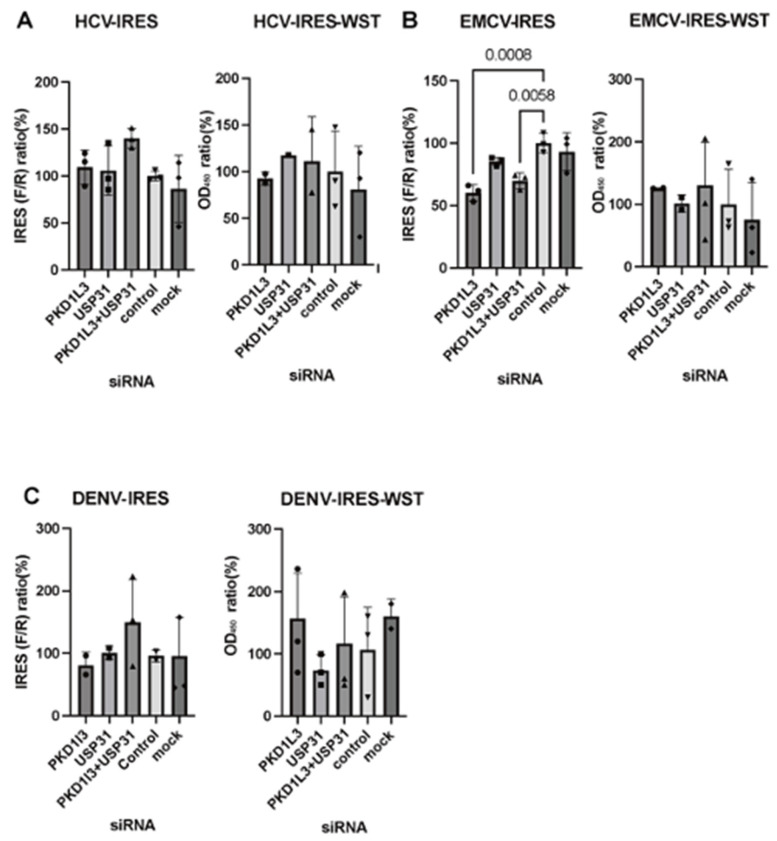
Effects of silencing *PKD1L3* and *USP31* on hepatitis C virus (HCV), encephalomyocarditis virus (EMCV), and dengue virus (DENV) internal ribosomal entry sites (IRESs). *p*-values less than 0.05, denoting significance, are shown. (**A**) *PKD1L3* and *USP31* siRNAs were transfected into HCV-IRES-co-transfected cells. IRES activity was calculated as a percentage relative to that in cells treated with control siRNA (left). The cell viability in the siRNA-treated groups was detected by performing WST assays (right). The percentage relative to that in the control siRNA-treated cells is indicated. Error bars are standard deviations (SDs; n = 3). (**B**) siRNAs targeting *PKD1L3* and *USP31* were transfected into EMCV-IRES-transfected cells. IRES activity was calculated as a percentage relative to that in control siRNA-treated cells (left). *p*-values showing a significant decrease (<0.05) are indicated. The cell viability of siRNA-treated cells was detected by performing WST assays (right). The percentage relative to that in the cells treated with control siRNA (%) is indicated. Error bars are SDs (n = 3). (**C**) siRNAs targeting *PKD1L3* and *USP31* were transfected into DENV-IRES-transfected cells. IRES activity was calculated as a percentage relative to that in cells treated with control siRNA (left). The cell viability in siRNA-treated groups was detected by performing WST assays (right). The percentage relative to that in the cells treated with the control siRNA (%) is indicated. Error bars are SDs (n = 3). F/R, ratio of firefly luciferase activity to *Renilla* luciferase activity. All experiments (n = 3) were performed in triplicate, and representative results are shown.

**Figure 7:**
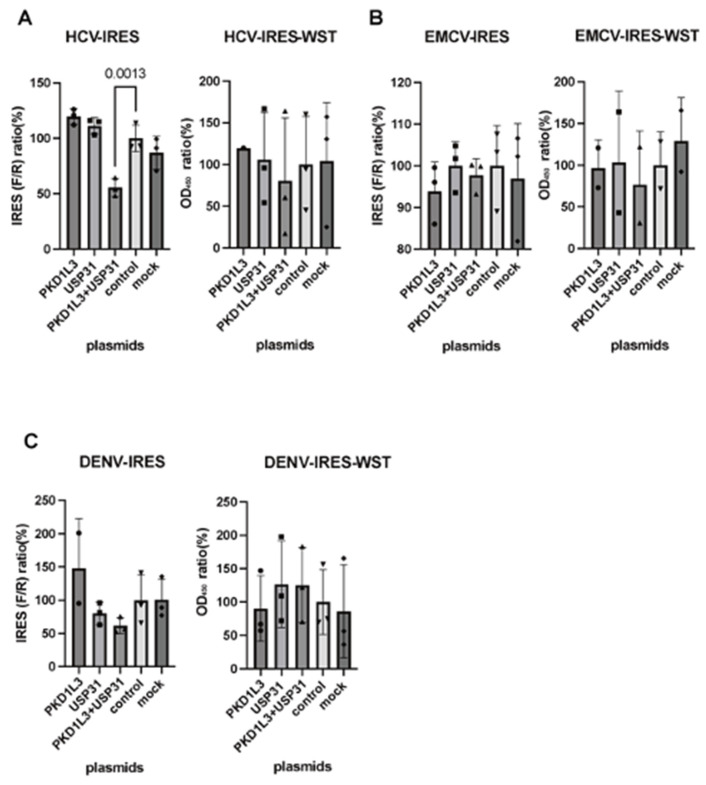
Effects of PKD1L3 and USP31 overexpression on hepatitis C virus (HCV), encephalomyocarditis virus (EMCV), and dengue virus (DENV) internal ribosomal entry sites (IRESs). (**A**) Effects of PKD1L3 and USP31 overexpression on HCV IRES. IRES activity (left) and cell viability (right) were measured as ratios relative to those in control vector-transduced cells. *p*-values less than 0.05 were considered significant and are indicated. The effects of PKD1L3 and USP31 overexpression on the EMCV IRES (**B**) and DENV IRES (**C**) were examined (left). The cell viability in siRNA-treated groups was detected by performing WST assays (right). The ratio relative to that in the control vector-transfected cells (%) is indicated. Error bars are standard deviations (n = 3). F/R, ratio of firefly luciferase activity to *Renilla* luciferase activity.

Neither *PKD1L3* nor *USP31* silencing significantly affected HCV-IRES or DENV-IRES activity. The only significant effects (reduced activity) in these assays were observed after silencing *PKD1L3* alone and combined silencing of *PKD1L3* and *USP31* in EMCV-IRES-expressing cells (Figure 6A–C). Neither PKD1L3 nor USP31 overexpression had a significant effect on EMCV- or DENV-IRES activity, and the only significant result (reduced activity) in these assays was observed for combined PKD1L3 and USP31 overexpression in HCV-IRES-expressing cells (Figure 7A–C).

## 4. Discussion

In the present study, we evaluated PKD1L3 and USP31 as host factors for various viral IRESs. This study provides further evidence for the interaction of these proteins with FMDV and CSFV IRESs, and to the best of our knowledge, this is the first investigation of their potential participation in the IRES-mediated translation of HCV, EMCV, and DENV. As key findings pertaining to FMDV and CSFV IRESs, our findings provide further confirmation of the roles of PKD1L3 and USP31 as host factors (silencing these proteins suppressed IRES activity). These results are in line with expectations based on a previous study in which pycnogenol (PYC) suppressed IRES activity and downregulated *PKD1L3* and *USP31* expression [18], which is consistent with our previous investigation of PKD1L3 and USP31 in IRES-expressing cells based on these two viruses [18]. Additionally, we found that *PKD1L3* silencing suppressed FMDV-, EMCV (Type I)-, and CSFV (Type IV)-IRES activity more strongly than that with *USP31* silencing, which is consistent with a previous microarray analysis of PYC-treated cells in which PKD1L3 expression was more strongly induced than USP31 expression (Table 1) [18]. The silencing of *PKD1L3* had a less pronounced effect on flaviviral IRESs (HCV and DENV), suggesting a differential role for PKD1L3 in IRESs. In line with the results of our previous studies [18,19], we found that overexpression had no significant effect on FMDV-IRES activity, suggesting that PKD1L3 and USP31 may have reached saturation in FMDV-IRES-expressing cells, whereas USP31 overexpression increased CSFV-IRES activity, suggesting that saturation was not reached for this protein in CSFV-IRES-expressing cells. However, because we did not directly assess protein occupancy of the IRES or perform biochemical assays, such as RNA pulldown or CLIP, to confirm saturation, the saturation hypothesis remains a plausible but unverified explanation of the observed differences. Further studies are required to directly evaluate the binding dynamics and functional limitations of these host factors based on viral IRESs. The only significant effect observed was a reduction in IRES activity following the combined overexpression of PKD1L3 and USP31 in HCV-IRES-expressing cells, which may be attributable to cytotoxic effects. However, dose-dependent analyses and additional specificity controls are required to validate these findings.

Our findings revealed distinct roles for PKD1L3 and USP31 in regulating IRES-mediated translation among the viruses examined. For the EMCV IRES, PKD1L3 silencing led to a significant reduction in IRES activity, suggesting that PKD1L3 may have a regulatory or facilitative function in EMCV-driven translation. This effect was also evident when *PKD1L3* knockdown was combined with *USP31* silencing, supporting the potential cooperative or compensatory interaction between these host factors in EMCV-IRES functions. Notably, PKD1L3 forms a complex with PKD2-L1, a member of the polycystic TRP subfamily involved in Ca^2+^ influx and sensory signaling pathways [29]. The PKD1L3–PKD2L1 complex has been implicated in various physiological responses, including sour taste reception [30], indicating that PKD1L3 may influence viral IRES activity through Ca^2+^-dependent signaling mechanisms or membrane-associated processes [31,32,33]. The potential of viral IRES-dependent translation as a therapeutic target has been demonstrated in HCV model systems by using several experimental approaches [34,35].

However, our analyses of the HCV IRES indicated that neither PKD1L3 nor USP31 play a major role in regulating translation. Silencing these genes with siRNAs had minimal effects on IRES activity, and the only notable reduction was observed after the co-overexpression of PKD1L3 and USP31, likely due to cytotoxic effects or competition with other host factors such as eIF3c and Hsp90 [36]. Previous studies have suggested functional links between PKD1L3, USP31 and eIF3c/Hsp90 [19] and that both eIF3c and Hsp90 interact with the HCV IRES [36]. These observations imply that eIF3c and Hsp90, rather than PKD1L3 or USP31, may serve as common host factors utilized by multiple IRESs, including those of HCV, FMDV, and CSFV; this possibility should be confirmed in future studies.

Type II IRESs, such as those in FMDV and EMCV, depend heavily on host factors for translation initiation. They require canonical initiation factors (eIF2, eIF3, eIF4A, eIF4B, and eIF4G) and ITAFs (e.g., PCBP1/2 and PTB) to fold RNA and recruit the 40S ribosome to the location near the start codon. Meanwhile, translation proceeds without extensive scanning, and it is sensitive to the abundance and activity of host proteins [11]. Conversely, Type IV IRESs, such as the HCV IRES, are largely structurally autonomous and directly engage the 40S ribosome and eIF2/eIF3 to position the AUG codon with minimal reliance on eIF4G, eIF4A, or ITAFs [11]. Notably, the diversity of viral IRESs is further augmented by Type VI IRESs (e.g., cricket paralysis virus), which can initiate translation from a non-AUG start codon without the use of an initiator Met-tRNA or any canonical initiation factors [11,37,38]. Furthermore, Type VI IRESs (e.g., cricket paralysis virus-IRES) can initiate translation from a non-AUG codon without using the initiator Met-tRNA or any canonical initiation factors [7,9,39], illustrating the diversity of IRESs. Similarly, DENV-IRES activity remained largely unaffected by either the silencing or overexpression of PKD1L3 and USP31, indicating that its translation depends on a distinct set of host factors.

Viruses can exploit the host ubiquitination machinery to modulate NF-κB signaling and facilitate infection [39]. USP31, a member of the large cysteine protease family of deubiquitinating enzymes [40], localizes to post-synaptic lipid rafts [41] and has been implicated in the regulation of NF-kB signaling [40,42] and tumor progression [42,43,44]. Although USP31 has been associated with cellular stress responses and immune signaling pathways relevant to viral infection, our findings indicate that it does not directly influence DENV-IRES-driven translation. Elucidating the regulatory mechanisms that control IRES activity during viral infection will deepen our understanding of how viruses manipulate the host translational machinery and may inform the development of targeted antiviral strategies. These results highlight virus-specific differences in host factor utilization; PKD1L3 plays a more prominent role in EMCV-IRES activity, whereas HCV and DENV IRESs appear to depend on alternative host interactions, including those involving eIF3c and Hsp90. Understanding the regulatory mechanisms that control IRES activity during viral infection will deepen our knowledge of how viruses manipulate the host translational machinery and may guide the development of targeted antiviral strategies.

## 5. Conclusions

Our findings suggest that PKD1L3 and/or USP31 play a role in CSFV-IRES-, FMDV-IRES-, and EMCV-IRES-mediated translation; however, their effects on HCV- or DENV-IRES activity appear less pronounced. Further investigations are needed to clarify the biological roles of these proteins in vivo during CSFV, FMDV, and EMCV infections.

## Figures and Tables

**Table 1:** Effects of PKD1L3 and USP31 modulation on viral internal ribosomal entry site (IRES) activity.

Factor	Gene Level	FMDV	CSFV	EMCV	HCV	DENV
PKD1L3	Normal	¯	¯	¯	¯	¯
	Silencing	Significantly suppresses IRES activity of FMDV IRES	Significantly suppresses IRES activity of CSFV IRES	Significantly suppresses IRES activity of EMCV IRES	No significant effect	No significant effect
	Overexpression	No significant effect	No significant effect	No significant effect	No significant effect	No significant effect
USP31	Normal	¯	¯	¯	¯	¯
	Silencing	No significant effect	No significant effect	No significant effect	No significant effect	No significant effect
	Overexpression	No significant effect	No significant effect	No significant effect	No significant effect	No significant effect
PKD1L3+USP31	Normal	¯	¯	¯	¯	¯
	Silencing	Significantly suppresses IRES activity of FMDV IRES	Significantly suppresses IRES activity of CSFV IRES	Significantly suppresses IRES activity of EMCV IRES	No significant effect	No significant effect
	Overexpression	No significant effect	No significant effect	No significant effect	Significantly suppresses IRES activity of HCV IRES	No significant effect

Footnote: ¯ = baseline IRES activity with normal expression.

## Data Availability

The original contributions presented in this study are included in the article/Supplementary Material. Further inquiries can be directed to the corresponding author.

## Supplementary Materials

The following supporting information can be downloaded from https://www.mdpi.com/article/10.3390/vetsci12121128/s1. Figure S1: A. The expression of GFP and Myc in the nucleus (DAPI) upon transfection with pRF-EGFP-PURO-EF1A-hPKD1L3-Myc was examined using an immunofluorescence assay (×400). B. The expression of GFP and USP31 in the nucleus (DAPI) upon transfection with pRF-EGFP-PURO-EF1A-hUSP31-HA was examined using an immunofluorescence assay (×400). Table S1. Quantification of PKD1L3 and USP31 nuclear localization was performed using Pearson’s correlation coefficient (ImageJ software).

## Abbreviations

The following abbreviations are used in this manuscript:

CSFV	Classical swine fever virus
DENV	Dengue virus
EMCV	Encephalomyocarditis virus
FMDV	Foot-and-mouth disease virus
GFP	Green fluorescent protein
HCV	Hepatitis C virus
HEK293	Human Embryonic Kidney 293 cells
Hsp90β	Heat shock protein 90 beta
eIF2	Eukaryotic initiation factor 2
eIF3	Eukaryotic initiation factor 3
eIF3c	Eukaryotic initiation factor 3 subunit c
eIF4A	Eukaryotic initiation factor 4A
eIF4B	Eukaryotic initiation factor 4B
eIF4G	Eukaryotic initiation factor 4G
IRES	Internal ribosome entry site
ITAFs	IRES-transacting factors
NF-κB	Nuclear factor kappa-light-chain-enhancer of activated B cells
OD	Optical density
PKD1L3	Polycystic kidney disease1 like 3
PCBP1/2	Poly rC-binding proteins 1 and 2
PTB	Polypyrimidine tract-binding protein
PYC	Pycnogenol
RBPs	RNA-binding proteins
siRNA	Small interfering RNA
USP31	Ubiquitin-specific peptidase 31
UTR	Untranslated region
