# Supplementary material for: Comparison of the Regulatory Effects of Host Factors on Viral Internal Ribosomal Entry Sites
Source: Vet Sci. 2025 Nov 27;12(12):1128. doi: 10.3390/vetsci12121128 (PMC12737507; doi:10.3390/vetsci12121128)
Supplement: Supplementary file 1 [file vetsci-12-01128-s001.zip › vetsci-3935392-supplementary.pdf]

A

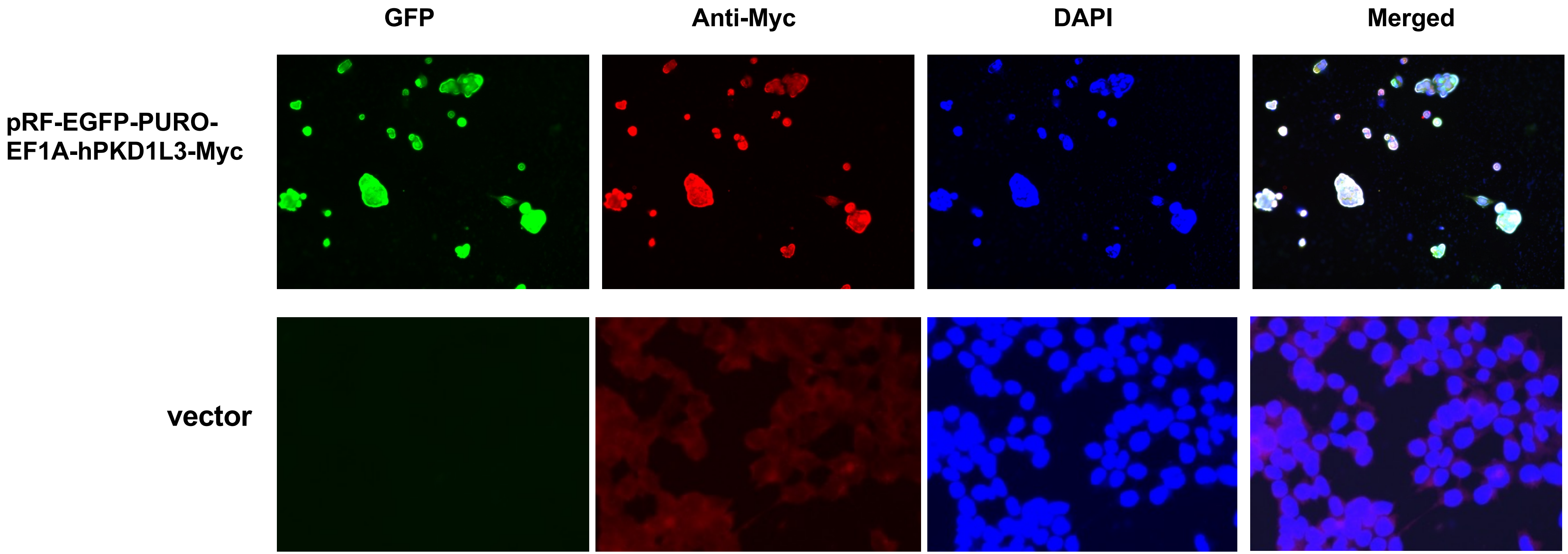
GFP
Anti-Myc
DAPI
Merged
pRF-EGFP-PURO-
EF1A-hPKD1L3-Myc
vector

Fig.S1

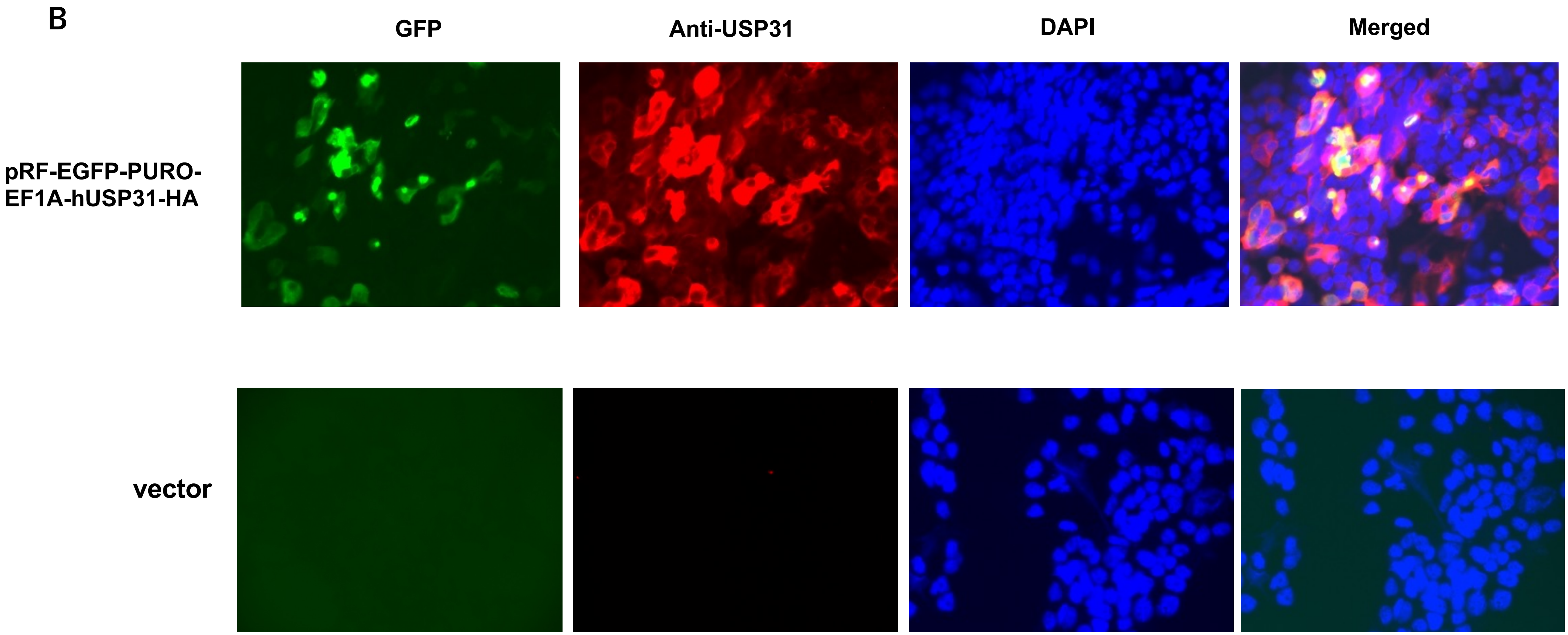
B
GFP
Anti-USP31
DAPI
Merged
pRF-EGFP-PURO-
EF1A-hUSP31-HA
vector

Fig.S1

Quantification of nuclear localization using Pearson's coefficient (Image-J software analysis)

Table S1A: pRF-EGFP-PURO-EF1A-hPKD1L3-Myc

Cell	GFP-GREEN	DAPI-Blue	ROI	Auto Threshold A	Auto Threshold B	GFP-nuclear localization Pearson's Coefficient
1	C1-Composite T1	C2-Composite T1	0001-0057-0023	Otsu	Otsu	0.963
2	C1-Composite T1	C2-Composite T1	0001-0037-0014	Otsu	Otsu	0.923
3	C1-Composite T1	C2-Composite T1	0001-0065-0104	Otsu	Otsu	0.852
4	C1-Composite T1	C2-Composite T1	0001-0119-0037	Otsu	Otsu	0.746
5	C1-Composite T1	C2-Composite T1	0001-0050-0138	Otsu	Otsu	0.975
6	C1-Composite T1	C2-Composite T1	0001-0046-0063	Otsu	Otsu	0.915
7	C1-Composite T1	C2-Composite T1	0001-0147-0139	Otsu	Otsu	0.869
8	C1-Composite T1	C2-Composite T1	0001-0141-0037	Otsu	Otsu	0.928
**Average**						**0.896375**

Cell	anti-myc PKD1L3_Red	DAPI-Blue	ROI	Auto Threshold A	Auto Threshold B	anti-myc PKD1L3 nucelar localization Pearson's Coefficient
1	C1-Composite T1	C2-Composite T1	0001-0047-0064	Otsu	Otsu	0.876
2	C1-Composite T1	C2-Composite T1	0001-0057-0022	Otsu	Otsu	0.966
3	C1-Composite T1	C2-Composite T1	0001-0038-0014	Otsu	Otsu	0.722
4	C1-Composite T1	C2-Composite T1	0001-0045-0130	Otsu	Otsu	0.693
5	C1-Composite T1	C2-Composite T1	0001-0050-0138	Otsu	Otsu	0.902
6	C1-Composite T1	C2-Composite T1	0001-0065-0104	Otsu	Otsu	0.765
7	C1-Composite T1	C2-Composite T1	0001-0141-0037	Otsu	Otsu	0.842
8	C1-Composite T1	C2-Composite T1	0001-0119-0037	Otsu	Otsu	0.718
9	C1-Composite T1	C2-Composite T1	0001-0032-0096	Otsu	Otsu	0.747
**Average**						**0.803444444**
					overall average	0.81

Table S1B: pRF-EGFP-PURO-EF1A-hUSP31-HA

Cell	GFP-GREEN	DAPI-Blue	ROI	Auto Threshold A	Auto Threshold B	GFP-nuclear localization Pearson's Coefficient
2	C1-Composite T1	C2-Composite T1	0001-0013-0063	Otsu	Otsu	-0.208
3	C1-Composite T1	C2-Composite T1	0001-0053-0052	Otsu	Otsu	-0.318
4	C1-Composite T1	C2-Composite T1	0001-0122-0049	Otsu	Otsu	0.122
5	C1-Composite T1	C2-Composite T1	0001-0108-0074	Otsu	Otsu	-0.117
6	C1-Composite T1	C2-Composite T1	0001-0116-0079	Otsu	Otsu	-0.38
7	C1-Composite T1	C2-Composite T1	0001-0100-0110	Otsu	Otsu	0.345
8	C1-Composite T1	C2-Composite T1	0001-0095-0148	Otsu	Otsu	-0.292
9	C1-Composite T1	C2-Composite T1	0001-0106-0135	Otsu	Otsu	0.122
10	C1-Composite T1	C2-Composite T1	0001-0098-0185	Otsu	Otsu	0.016
11	C1-Composite T1	C2-Composite T1	0001-0155-0068	Otsu	Otsu	-0.087
12	C1-Composite T1	C2-Composite T1	0001-0081-0082	Otsu	Otsu	-0.08
13	C1-Composite T1	C2-Composite T1	0001-0068-0106	Otsu	Otsu	-0.273
14	C1-Composite T1	C2-Composite T1	0001-0047-0139	Otsu	Otsu	0.347
Average						-0.061769231

Cell	anti-USP31-Red	DAPI-Blue	ROI	Auto Threshold A	Auto Threshold B	USP31-nuclear localization Pearson's Coefficient
1	C1-Composite T1	C2-Composite T1	0001-0012-0063	Otsu	Otsu	-0.411
2	C1-Composite T1	C2-Composite T1	0001-0078-0050	Otsu	Otsu	-0.427
3	C1-Composite T1	C2-Composite T1	0001-0131-0026	Otsu	Otsu	-0.042
4	C1-Composite T1	C2-Composite T1	0001-0115-0078	Otsu	Otsu	-0.218
5	C1-Composite T1	C2-Composite T1	0001-0156-0067	Otsu	Otsu	-0.024
6	C1-Composite T1	C2-Composite T1	0001-0145-0104	Otsu	Otsu	0.185
7	C1-Composite T1	C2-Composite T1	0001-0045-0141	Otsu	Otsu	0.074
8	C1-Composite T1	C2-Composite T1	0001-0064-0186	Otsu	Otsu	0.59
9	C1-Composite T1	C2-Composite T1	0001-0066-0126	Otsu	Otsu	-0.508
10	C1-Composite T1	C2-Composite T1	0001-0121-0049	Otsu	Otsu	0.325
11	C1-Composite T1	C2-Composite T1	0001-0133-0238	Otsu	Otsu	-0.468
12	C1-Composite T1	C2-Composite T1	0001-0093-0249	Otsu	Otsu	-0.507
13	C1-Composite T1	C2-Composite T1	0001-0137-0062	Otsu	Otsu	-0.252
14	C1-Composite T1	C2-Composite T1	0001-0055-0213	Otsu	Otsu	-0.591
15	C1-Composite T1	C2-Composite T1	0001-0098-0057	Otsu	Otsu	-0.313
16	C1-Composite T1	C2-Composite T1	0001-0070-0022	Otsu	Otsu	-0.447
17	C1-Composite T1	C2-Composite T1	0001-0167-0031	Otsu	Otsu	-0.08
Average						-0.183176471
					overall average	-0.18

*nuclear/cytoplasmic localization of PKD1L3 and USP31 was calculated based on the Fig.S1 using ImageJ software.
